# Physical control and coordination in childhood and adult obesity: longitudinal birth cohort study

**DOI:** 10.1136/bmj.a699

**Published:** 2008-08-12

**Authors:** Walter Osika, Scott M Montgomery

**Affiliations:** 1 Department of Cardiology, Örebro University Hospital, SE-701 85 Örebro, Sweden; 2 Clinical Research Centre, Örebro University Hospital; 3 Department of Primary Care and Social Medicine, Charing Cross Hospital, Imperial College, London; 4 Clinical Epidemiology Unit, Department of Medicine at Karolinska University Hospital, Karolinska Institutet, Stockholm, Sweden

## Abstract

**Objective** To identify whether measures of childhood physical control and coordination as markers of neurological function are associated with obesity in adults.

**Design** Longitudinal birth cohort study.

**Setting** National child development study in Great Britain.

**Participants** 11?042 people born during one week in 1958.

**Main outcome measure** Obesity at age 33 years defined as body mass index =30.

**Results** Among 7990 cohort members at age 7 years, teachers reported that poor hand control, poor coordination, and clumsiness “certainly applied” more often among those who would be obese adults, producing adjusted odds ratios of 1.57 (95% confidence interval 1.13 to 2.20; P=0.008) for poor hand control, 2.30 (1.52 to 3.46; P<0.001) for poor coordination, and 3.91 (2.61 to 5.87; P<0.001) for clumsiness. Among 6875 participants who had doctor administered assessments with continuous scores at age 11 years, poorer function was associated with later obesity, indicated by adjusted odds ratios (change in risk per unit increase in score) of 0.88 (0.81 to 0.96; P=0.003) for copying designs, 0.84 (0.78 to 0.91; P<0.001) for marking squares, and 1.14 (1.06 to 1.24; P<0.001) for picking up matches (a higher score indicates poor function in this test). Further adjustment for contemporaneous body mass index at age 7 or 11 years did not eliminate statistical significance for any of the associations.

**Conclusion** Some aspects of poorer neurological function associated with adult obesity may have their origins in childhood.

## Introduction

Obesity in adults is associated with cognitive impairment and dementia,^1^ ^2^ ^3^ and this is assumed to be a consequence of obesity or obesity related processes such as secretion of bioactive hormonal compounds, altered insulin signalling, diabetes, hypertension, and other cardiovascular disease processes.^4^ ^5^ ^6^ ^7^ ^8^ ^9^ However, some relevant processes begin in earlier life, potentially with different mechanisms.

Obese adults and those with adult onset type 2 diabetes mellitus may already have lower levels of cognitive function in childhood, consistent with a subtle developmental impairment.^10^ ^11^ Therefore, pathways to impaired cognitive function in these groups may begin much earlier in life than was previously thought, reflecting early biological processes relevant to neurological function and cognition. An alternative explanation is that tests of cognitive function are influenced directly by social and cultural factors,^12^ ^13^ rather than indicating a neurological mechanism, and these social influences also increase future risk of obesity.

We investigated whether tests of physical control and coordination in children are associated with obesity in adults, by using measures less subject to confounding by social factors than many tests of cognition. We focused on measures of fine hand control and avoided assessments more likely to be influenced by participation in sports, such as catching balls or balance. We used British longitudinal data to examine the association of tests in childhood with obesity at age 33. Associations of these childhood tests with obesity in later life will emphasise the importance of a life course approach in understanding some aspects of poorer neurological function in obesity.

## Methods

The national child development study is following everyone born between 3 and 9 March 1958 and living in Great Britain, with data collection sweeps at various ages throughout childhood and in adult life.^14^ The study was originally of approximately 17?000 births, but the subsequent exclusion of Northern Ireland, death, emigration, and other causes of attrition reduced the sample size, although the cohort has remained broadly representative of the target population.^15^ A total of 11?042 people had information on body mass index at age 33; among these, 7990 also had information for assessments and potential confounding factors at age 7 and 6875 had such information at age 11 and were free from major disabilities and in mainstream schools.

At birth, midwives recorded information on sex, birth weight in ounces, gestational age in weeks, mother’s age, maternal smoking during the fourth month of pregnancy (non-smokers, medium, variable, and heavy smokers). Social class (Registrar General) based on the father’s occupation was categorised as I, II, III non-manual, III manual, IV, V, and “not assigned” (where the father was not present or not working).

At age 7, classroom teachers (who also supervised physical education classes) were asked to identify poor ability in hand control, coordination, and overall clumsiness, scored as certainly, somewhat, no, and unsure. The Bristol social adjustment guide is a test in which class teachers recorded descriptions of behaviour that applied to the child among 150 items. The overall score has been useful in epidemiological surveys as an indicator of behavioural maladjustment and deviant behaviour.^16^ A local authority medical officer measured weight and height and recorded information on mental retardation and all significant disabilities and chronic illnesses.

At age 11 years, local authority medical officers measured height and weight, as well as administering a series of functional assessments, of which we selected three for these analyses as indicating hand control and coordination. We selected tests that use the right hand, as this is the dominant hand for most people. Laterality is not associated with obesity and so cannot be a confounding factor. The first test involved copying a simple design, and the accuracy of the copy was scored from 0 to 12. The second test involved marking squares on paper within one minute (maximum 200). The third test recorded the time in seconds that it took to pick up 20 matches (maximum 99 s); unlike for the other tests, a higher score indicated poorer performance. Pubertal development was assessed by using scores for breast development and pubic hair assessments in girls and genitalia development and pubic hair in boys (0-5 for each measure). The scores were summed for boys and girls separately and were then converted into standard deviation units and combined. General ability (cognitive function) tests with a range of 0-76 were administered at school.^17^

At age 33, trained interviewers measured height and weight (the latest measured rather than self reported records); obesity was defined as body mass index (weight (kg)/(height (m)^2^) of 30 or over. Ethnic origin was categorised as white British (more than 96%), Irish, white other, white and black Caribbean, white and black African, white and Asian, other mixed race, Indian, other Asian, Caribbean, African, other black, or other ethnic group.

### Statistical analysis

We used logistic regression to estimate associations with obesity at age 33 as the dependent variables in six separate models for each of the measures of physical control and coordination at ages 7 and 11 years. We adjusted these models for social class, sex, mother’s age, birth weight standardised for gestational age, ethnic origin, maternal smoking during pregnancy, and the four chronic disease variables associated with either later obesity or motor function (general motor disability, mental retardation, epilepsy, and other central nervous system conditions). We included Bristol social adjustment guide score in the models for tests at age 7 and the puberty score for age 11. Further adjustment was for body mass index (weight (kg)/(height (m)^2^) at age 7 for tests at age 7 and body mass index (weight (kg)/(height (m)^2^ or (weight (kg)/(height (m)^3^) at age 11 years and general (cognitive) ability score in the models relevant to tests at age 11. All measures were categorical and were modelled as series of binary dummy variables, except Bristol social adjustment guide score, childhood body mass index, puberty score, and general ability score. We used SPSS for Windows version 16 for the analyses.

## Results

Table 1 shows the distribution of obesity at age 33 by childhood social class and sex overall and in the subsets with complete data at ages 7 and 11, to illustrate the effects on the cohort of non-participation. Lower social class was associated with increased risk of obesity. Compared with the more complete sample, the social class and sex distributions for the age 7 and age 11 analyses are broadly representative; the only notable change was for the small group without an assigned social class.

**Table 1 tbl1:** Childhood characteristics and obesity at age 33 years in complete sample and subsets used in analyses for tests at ages 7 and 11 years. Values are numbers (percentages)

Characteristic	Obesity at age 33 years		
	Complete sample (n=11 042)	Age 7 year subset (n=7990)	Age 11 year subset (n=6875)
Social class at birth:			
I	35/474 (7.4)	21/355 (5.9)	16/305 (5.2)
II	92/1378 (6.7)	72/1071 (6.7)	59/937 (6.3)
III non-manual	93/1030 (9.0)	67/792 (8.5)	59/693 (8.5)
III manual	654/5074 (12.9)	502/3930 (12.8)	425/3380 (12.6)
IV	174/1208 (14.4)	127/892 (14.2)	106/786 (13.5)
V	113/836 (13.5)	85/623 (13.6)	66/503 (13.1)
Not assigned*	104/1042 (10.0)	38/327 (11.6)	33/271 (12.2)
Sex:			
Male	595/5431 (11.0)	425/3930 (10.8)	352/3365 (10.5)
Female	670/5611 (11.9)	487/4060 (12.0)	412/3510 (11.7)

*Includes mothers without partners and those who were not working or whose employment could not be classified.

Table 2 shows the associations of teacher assessed hand control, coordination, and clumsiness at age 7 with obesity at age 33, estimated with logistic regression. All three measures showed a graded and statistically significant association of poorer control and coordination with obesity at age 33, and the associations were independent of the markers of childhood circumstances and behaviour. Further adjustment for body mass index at age 7 did not eliminate statistical significance.

**Table 2 tbl2:** Teachers’ assessments at age 7 years and obesity at age 33 years

Assessments	Obesity (%) at age 33 years			Unadjusted			Adjusted*			Adjusted†	
	No (n=7078)	Yes (n=912)		Odds ratio (95% CI)	P value		Odds ratio (95% CI)	P value		Odds ratio (95% CI)	P value
Poor hand control:											
Certainly	244 (3.4)	52 (5.7)		1.83 (1.34 to 2.49)	<0.001		1.57 (1.13 to 2.20)	0.008		1.48 (1.04 to 2.10)	0.030
Somewhat	998 (14.1)	177 (19.4)		1.52 (1.27 to 1.82)	<0.001		1.38 (1.14 to 1.67)	0.001		1.33 (1.09 to 1.63)	0.005
No	5824 (82.3)	679 (74.5)		Reference			Reference			Reference	
Unsure	12 (0.2)	4 (0.4)		2.86 (0.92 to 8.89)	0.070		2.69 (0.85 to 8.48)	0.091		1.99 (0.60 to 6.58)	0.275
Poor coordination:											
Certainly	123 (1.7)	38 (4.2)		2.58 (1.78 to 3.74)	<0.001		2.30 (1.52 to 3.46)	<0.001		1.57 (1.01 to 2.45)	0.047
Somewhat	671 (9.5)	118 (12.9)		1.47 (1.19 to 1.81)	<0.001		1.37 (1.10 to 1.71)	0.005		1.16 (0.92 to 1.47)	0.223
No	6249 (88.3)	748 (82.0)		Reference			Reference			Reference	
Unsure	35 (0.5)	8 (0.9)		1.91 (0.88 to 4.13)	0.100		1.75 (0.80 to 3.83)	0.159		1.50 (0.67 to 3.38)	0.329
Clumsy:											
Certainly	95 (1.3)	43 (4.7)		4.05 (2.80 to 5.86)	<0.001		3.91 (2.61 to 5.87)	<0.001		2.04 (1.31 to 3.20)	0.002
Somewhat	659 (9.3)	162 (17.8)		2.20 (1.82 to 2.66)	<0.001		2.24 (1.82 to 2.76)	<0.001		1.51 (1.21 to 1.89)	<0.001
No	6291 (88.9)	703 (77.1)		Reference			Reference			Reference	
Unsure	33 (0.5)	4 (0.4)		1.09 (0.38 to 3.07)	0.878		1.04 (0.36 to 2.97)	0.947		1.00 (0.34 to 2.93)	0.996

*Adjusted for social class, sex, mother’s age, birth weight standardised for gestational age, ethnic origin, maternal smoking during pregnancy, Bristol social adjustment guide score, and four chronic disease variables associated with either later obesity or motor function (general motor disability, mental retardation, epilepsy, and other central nervous system conditions).

†Adjusted for all above measures and also for body mass index at age 7.

Table 3 shows the association of doctor administered tests of hand control and coordination at age 11 with obesity at age 33. The tests scores are continuous measures, so no reference category exists; the odds ratios are modest in magnitude, as they represent the increase in risk of obesity associated with a one unit change in the score. The significantly lower scores (worse performance) for copying designs and marking squares associated with obesity are illustrated by odds ratios below 1.00. In contrast to the other tests, a higher score in the picking up matches test indicates worse performance (a longer time); this was significantly associated with obesity, indicated by odds ratios above 1.00. Adjustment for childhood characteristics and pubertal development did not notably alter the associations or their statistical significance. Further adjustment for body mass index at age 11 did not eliminate statistical significance for associations of obesity at age 33 with the tests, although it became borderline for picking up matches. Altering the measure of body mass index at age 11 years to weight (kg)/(height (m)^3^ did not notably change any of the results (data not shown), except for picking up matches, where the effect of adjustment was less pronounced (odds ratio 1.10, 95% confidence interval 1.01 to 1.19; P=0.03).

**Table 3 tbl3:** Assessments done during medical examination at age 11 years and risk of obesity at age 33 years

Assessment	Unadjusted			Adjusted*			Adjusted†	
	Odds ratio (95% CI)	P value		Odds ratio (95% CI)	P value		Odds ratio (95% CI)	P value
Copying designs	0.84 (0.77 to 0.91)	<0.001		0.88 (0.81 to 0.96)	0.003		0.88 (0.81 to 0.97)	0.006
Marking squares	0.83 (0.77 to 0.90)	<0.001		0.84 (0.78 to 0.91)	<0.001		0.82 (0.75 to 0.89)	<0.001
Picking up matches	1.17 (1.08 to 1.26)	<0.001		1.14 (1.06 to 1.24)	<0.001		1.09 (1.00 to 1.18)	0.050

Independent measures are continuous test scores; modest magnitude of odds ratios reflects change in obesity risk associated with one unit change in test score.

*Adjusted for social class, sex, mother’s age, birth weight standardised for gestational age, ethnic origin, maternal smoking during pregnancy, pubertal development, and four chronic disease variables associated with either later obesity or motor function (general motor disability, mental retardation, epilepsy, and other central nervous system conditions).

†Adjusted for all above measures and also for body mass index at age 11.

Including the total (cognitive) ability score in the adjusted models for the association of age 11 scores with obesity at age 33 may represent an over-adjustment, as both the cognitive function score and physical control score may be relevant to neurological function. After this adjustment, the association of obesity with copying designs was not statistically significant: 0.94 (0.86 to 1.01; P=0.116). However, we found significant associations with obesity at age 33 for marking squares and picking up matches, which had odds ratios of 0.87 (0.80 to 0.94; P<0.001) and 1.13 (1.05 to 1.22; P=0.002).

We excluded the participants with a diagnosis of diabetes mellitus up to age 16 years (n=24), but none of the estimates was notably altered (data not shown). Exclusion of the minority with a non-British ethnic origin had almost no effect on the results.

## Discussion

All the investigated measures of physical control and coordination at ages 7 and 11 were associated with obesity in adults, independent of multiple markers of social and material conditions in childhood. Even adjustment for contemporaneous childhood body mass index did little to alter the associations, as it was not strongly associated with the tests (data not shown), except picking up matches. This indicates that childhood body mass is not responsible for the differences in function seen. To ensure that this was not due to poor estimation of childhood body mass index, we also modelled an alternative estimate of childhood body mass index that may be a better indicator of childhood body mass.^18^ Arguably, including childhood body mass index is an over-adjustment that may mask important associations, as many (although far from all) obese adults tend to be heavier children.^19^ Some early life exposures or characteristics associated with obesity in adults may influence development of physical control and coordination long before the onset of obesity.

### Use of tests of motor competence

A disadvantage of this study is that it could not identify specific measures of neurological function; instead it used markers of physical control and coordination likely to be relevant. Unlike earlier studies of cognitive ability,^10^ ^11^ the functional assessments used here are less susceptible to confounding by immediate social factors, as poor performance in cognitive function tests may reflect a lack of knowledge and experience rather than indicating true cognition.^12^ Thus, the study provides evidence of poorer motor competence indicating neurological function in childhood among those who will be obese adults. Given the social gradient of obesity, social factors are likely to be of primary importance in explaining its causes and also the associated increased risk of poorer physical control and coordination.

As the assessments at age 7 were based on teachers’ observation of pupils, we included adjustment for Bristol social adjustment guide score to reduce the possibility that deviant behaviour influenced the teachers’ perceptions of children. The assessments at age 11 were perhaps more objective, and to avoid confounding by development associated with puberty we made adjustments for markers of pubertal stage. We found no evidence that a chronic disease or disability diagnosed in childhood explained the reported associations.

### Childhood body mass and cognitive function

Inclusion of childhood body mass index and the general (cognitive) ability score in the models clearly represents over-adjustment but also provides useful information. Adjustment for childhood body mass index not only indicates that body mass does not seem to directly influence physical control and coordination but also represents an indirect adjustment for a variety of factors, such as physical activity, that are plausible confounding factors. However, body mass index may be too crude a measure to identify specific types of adipose tissue in childhood that indicate physical activity or produce bioactive compounds.^2^ ^5^ ^20^ ^21^ The effect of adjustment for general ability indicates associations with cognitive function in copying designs. Interestingly, associations between obesity in adults and the other tests of physical control and coordination are more independent of cognitive function, so exclusive mediation through persistently lower cognitive ability is unlikely. The varying effects of adjustment for cognitive function and childhood body mass index depending on the test suggest heterogeneity in the childhood characteristics measured by these tests.

### Potential limitations and the importance of a life course approach 

Although the cohort studied is generally representative of the original cohort, attrition was greatest among more disadvantaged groups that also tend to contain a higher proportion of obese cohort members.^14^ This bias may result in more conservative estimates of the reported associations, as one extreme of the obesity distribution is attenuated, but is unlikely to create spurious associations. The reported associations are independent of several well recognised measures of socioeconomic and personal characteristics, as well as additional markers such as birth weight and maternal smoking during pregnancy, which may be relevant to risk of obesity but also signals a variety of associated cultural and economic exposures.^22^ ^23^ ^24^ Many other environmental or individual characteristics could explain the associations. These might be considered as confounding factors and are important targets for future research to assist in our understanding of how risks for obesity and associated complications accumulate across the life course. Clearly, diet and exercise may be important, as continuity in patterns of physical activity are likely to influence physical control and coordination as well as risk of obesity. Exercise may influence physical control and coordination, or people with lower motor competence may be less likely to exercise, thus increasing risk of obesity. Some aspects of personality (possibly also influenced by earlier environmental exposures) could be potentially relevant to behaviour influencing physical control and coordination as well as risk of obesity. Rather than being explained by a single factor, an accumulation throughout life of many associated cultural, personal, and economic exposures is likely to underlie the risks for obesity and some elements of associated neurological function.

### Conclusion

This study cannot identify the specific biological processes linking poorer physical control and coordination in childhood with later obesity. However, it suggests that some of the processes associated with poorer neurological function in obese adults have their origins in childhood.

**What is already known on this topic**

Poorer cognitive function in childhood is associated with obesity and type 2 diabetes in adults
Whether this is mediated through neurological function or confounding by social factors that limit performance in cognitive function tests is unclear

**What this study adds**

Poorer physical control and coordination in childhood is associated with obesity in adults
Some aspects of poorer neurological function associated with obesity in adults may have their origins in childhood

## Notes

Ethical approval: Not required for this analysis of anonymous data, although consent was initially obtained from parents before data collection and was subsequently sought from individual cohort members in later sweeps, including for access to medical records. Regional ethics committee approval was obtained for data collection involving medical examinations.

Provenance and peer review: Not commissioned; externally peer reviewed.

## Notes

**Cite this as:** *BMJ* 2008;337:a699

## References

[1] Whitmer RA, Gunderson EP, Barrett-Connor E, Quesenberry CP Jr, Yaffe K. Obesity in middle age and future risk of dementia: a 27 year longitudinal population based study. BMJ 2005;330:1360. 15863436 10.1136/bmj.38446.466238.E0 PMC558283

[2] Knecht S, Ellger T, Levine JA. Obesity in neurobiology. Prog Neurobiol 2008;84:85-103. 18006138 10.1016/j.pneurobio.2007.09.003

[3] Gustafson D. Adiposity indices and dementia. Lancet Neurol 2006;5:713-20. 16857578 10.1016/S1474-4422(06)70526-9

[4] Graf C, Koch B, Kretschmann-Kandel E, Falkowski G, Christ H, Coburger S, et al. Correlation between BMI, leisure habits and motor abilities in childhood (CHILT-project). Int J Obes Relat Metab Disord 2004;28:22-6. 14652619 10.1038/sj.ijo.0802428

[5] Yaffe K, Kanaya A, Lindquist K, Simonsick EM, Harris T, Shorr RI, et al. The metabolic syndrome, inflammation, and risk of cognitive decline. JAMA 2004;292:2237-42. 15536110 10.1001/jama.292.18.2237

[6] Craft S. Insulin resistance and Alzheimer’s disease pathogenesis: potential mechanisms and implications for treatment. Curr Alzheimer Res 2007;4:147-52. 17430239 10.2174/156720507780362137

[7] Strachan MW, Price JF, Frier BM. Diabetes, cognitive impairment, and dementia. BMJ 2008;336:6. 18174567 10.1136/bmj.39386.664016.BE PMC2174783

[8] Reitz C, Tang MX, Manly J, Mayeux R, Luchsinger JA. Hypertension and the risk of mild cognitive impairment. Arch Neurol 2007;64:1734-40. 18071036 10.1001/archneur.64.12.1734 PMC2672564

[9] Rosengren A, Skoog I, Gustafson D, Wilhelmsen L. Body mass index, other cardiovascular risk factors, and hospitalization for dementia. Arch Intern Med 2005;165:321-6. 15710796 10.1001/archinte.165.3.321

[10] Chandola T, Deary IJ, Blane D, Batty GD. Childhood IQ in relation to obesity and weight gain in adult life: the national child development (1958) study. Int J Obes 2006;30:1422-32. 10.1038/sj.ijo.0803279 16520812

[11] Olsson GM, Hulting AL, Montgomery SM. Cognitive function in children and subsequent type 2 diabetes mellitus. Diabetes Care 2008;31:514-6. 18083794 10.2337/dc08-0676 PMC2453642

[12] Jefferis BJ, Power C, Hertzman C. Birth weight, childhood socioeconomic environment, and cognitive development in the 1958 British birth cohort study. BMJ 2002;325:305. 12169505 10.1136/bmj.325.7359.305 PMC117769

[13] Tong S, Baghurst P, Vimpani G, McMichael A. Socioeconomic position, maternal IQ, home environment, and cognitive development. J Pediatr 2007;151:284-8. 17719939 10.1016/j.jpeds.2007.03.020

[14] Ferri E. Life at 33: the fifth follow-up of the national child development study. London: National Children’s Bureau, 1993.

[15] Power C, Elliott J. Cohort profile: 1958 British birth cohort (national child development study). Int J Epidemiol 2006;35:34-41. 16155052 10.1093/ije/dyi183

[16] Stott GH. The social adjustment of children: manual to the Bristol social adjustment guides. London: Hodder and Stoughton, 1987.

[17] Douglas JWB. The home and the school. London: MacGibbon and Kee, 1964.

[18] Power C, Lake JK, Cole TJ. Measurement and long-term health risks of child and adolescent fatness. Int J Obes 1997;21:507-26. 10.1038/sj.ijo.0800454 9226480

[19] Viner RM, Cole TJ. Adult socioeconomic, education, social and psychological outcomes of childhood obesity: a national birth cohort study. BMJ 2005;330:1354-8. 15901644 10.1136/bmj.38453.422049.E0 PMC558281

[20] Hausman DB, DiGirolamo M, Bartness TJ, Hausman GJ, Martin RJ. The biology of white adipocyte proliferation. Obes Rev 2001;2:239-54. 12119995 10.1046/j.1467-789x.2001.00042.x

[21] Yki-Järvinen H, Westerbacka J. Vascular actions of insulin in obesity. Int J Obes Relat Metab Disord 2000;24:S25-8. 10997603 10.1038/sj.ijo.0801272

[22] Montgomery SM, Ekbom A. Smoking during pregnancy and diabetes mellitus in a British longitudinal birth cohort. BMJ 2002;324:26-7. 11777801 10.1136/bmj.324.7328.26 PMC61655

[23] Toschke AM, Montgomery SM, Pfeiffer U, von Kries R. Early intrauterine exposure to tobacco-inhaled products and obesity. Am J Epidemiol 2003;158:1068-74. 14630602 10.1093/aje/kwg258

[24] Breslau N, Paneth N, Lucia VC, Paneth-Pollak R. Maternal smoking during pregnancy and offspring IQ. Int J Epidemiol 2005;34:1047-53. 16085682 10.1093/ije/dyi163

